# Race/Ethnicity, insurance, income and access to care: the influence of health status

**DOI:** 10.1186/1475-9276-12-29

**Published:** 2013-05-11

**Authors:** Tze-Fang Wang, Leiyu Shi, Xiaoyu Nie, Jinsheng Zhu

**Affiliations:** 1 School of Nursing, National Yang Ming University, Taipei 112, Taiwan; 2 Primary Care Policy Center, Johns Hopkins University, 624 N. Broadway, Baltimore, MD 21205, USA; 3 Bloomberg School of Public Health, Johns Hopkins University, 624 N. Broadway, Baltimore MD 21205, USA

**Keywords:** Access, Income, Insurance, Race/ethnicity, Health status

## Abstract

**Objectives:**

To examine health care access disparities with regard to health status and presence of functional limitations, a common measure of disability and multimorbidity, after controlling for individual’s race/ethnicity, insurance status and income in the U.S. using the latest survey data.

**Methods:**

Using data from the 2009 Family Core component of the National Health Interview Survey (NHIS), we examined six measures of access to care in the twelve months prior to the interview. Covariates included self-perceived health status and the presence of functional limitations, race/ethnicity, insurance status, income, and other socioeconomic characteristics. Multiple logistic regressions were used to examine the associations.

**Results:**

People with functional limitations or worse health status experience greater barriers to access. Insurance status was the single factor that was associated with all six measures of access. Disparities among racial/ethnic groups in most access indicators as well as income levels were insignificant after taking into account individuals’ health status measures.

**Conclusions:**

Interventions to expand insurance coverage and the Patient Protection and Affordable Care Act are expected to contribute to reducing disparities in access to care. However, to further improve access to care, emphasis must be placed on those with poorer health status and functional limitations.

## Introduction

The Institute of Medicine (IOM) defined disparity as “a difference in access or treatment provided to members of different racial or ethnic groups that is not justified by the underlying health conditions or treatment p of patients [1].” Access to health care services has been one of the major topic areas for both Healthy People 2010 and Healthy People 2020. In Healthy People 2010, it was one of the goals to eliminate racial/ethnic disparities in access to health care [2]. In Healthy People 2020, relevant goals included increasing the proportion of persons with a usual primary care provider, increasing the proportion of persons who have a specific source of ongoing care, reducing the proportion of individuals who are unable to obtain or delay in obtaining necessary medical care, dental care, or prescription medicines, etc [3].

Andersen (1995) proposed a framework of access to health care in which access is determined by environment including health care system and external environment, population characteristics which include predisposing characteristics such as demographic factors, community and personal enabling resources such as income and health insurance, and health needs which consist of both perceived and evaluated health status including the presence of comorbidity and disability [4]. Empirical studies have demonstrated that race/ethnicity, health insurance status, income, and demographic characteristics including health status are important contributing factors to access disparities [5,6].

The association between race/ethnicity and access to care has been studied for a long time. Although some studies reported no significant difference between non-Hispanic Whites and minority groups in access to certain preventive [7-10], mental health [11], and specialist services [12], a larger body of literature concluded that racial/ethnic disparities in access to care persist or have even worsened during the past few years [6-8,13-22]. In addition, the influence of insurance on access to care has been well established. Uninsured adults were less likely to get preventive care and physician care, and therefore, more likely to have unmet needs than insured individuals [23-26], which ultimately result in fundamental differences in health outcomes and overall life experiences [27]. According to Kenny (2012), uninsured population on average received only 55 percent of medical services and slightly more than a quarter of dental care of those insured. Furthermore, more than half of uninsured adults did not visit a doctor in 2010 [23]. Lastly, low income is another barrier to access to care [25,27-29]. However, research has pointed out that even among high-income adults, those uninsured still have significantly lower use of recommended health care services than the insured [30].

However, most of the prior research had not adequately examined or controlled for the impact of the patients’ health status such as multimorbidity and functional limitations. As is well-known, people of racial/ethnic minorities and low-income typically also have worse health status than their counterparts [31,32]. Without adjusting for health status, it is not clear whether observed racial and income-related disparities are stand-alone or a reflection of health disparities.

The purpose of this study is to examine health care access disparities with regard to health status and presence of functional limitations, a common measure of disability and multimorbidity, after controlling for individual’s race/ethnicity, insurance status and income in the U.S. using the latest survey data.

## Methods

### Data

Data for this study came from the Family Core component of the 2009 National Health Interview Survey (NHIS) conducted by the National Center for Health Statistics (NCHS) to monitor the health of the civilian noninstitutionalized population residing in the United States [33]. A multistage sampling strategy was employed and the sample was drawn from 50 States and the District of Columbia. Additionally, an oversampling strategy was applied to include a larger proportion of minority groups [33]. For the purpose of this study, we only used adult respondents who visited a doctor or other health care professional during the previous two weeks. The decision to limit the analysis to respondents with a recent health professional visit was based on the following considerations. First, since the access questions we used require respondents to remember their most recent experience, limiting the analysis to those with a recent health care visit would minimize recall bias. Second, there could be significant differences between those with health care experience and those without including health care needs and belief in addition to access issues. Therefore, our analysis that focused on those with health care experience could attenuate the likely influences of those factors. Of course we recognize that this decision could also underestimate the access barriers, i.e., excluding those who have not been successful in accessing health care.

### Measures

Six indicators of access to care in the twelve months prior to the interview were defined: (1) no usual place of care, (2) unable to get medical care, (3) delayed medical care, (4) unable to get dental care, (5) unable to get mental health care, and (6) unable to get prescription drugs. These indicators represented commonly used measures of access to care and are available in the NHIS.

The major independent variables are the presence of functional limitations and self-perceived general health status (excellent/very good/good and fair/poor). Functional limitations were defined as restrictions in one or more domains of functional physical and mental actions [4,34]. It is well-known that functional disability is significantly associated with the presence of multimorbidity and disability as functional status deteriorates with the increase of chronic conditions [35-38]. Specific diagnosis of certain conditions, including CHF, diabetes and/or chronic respiratory disease, have been proved to be able to predict decline in functional status [39]. Since functional limitations and self-perceived general health status are significantly correlated, we assessed whether multicollinearity exists in a regression model with both measures included. However, due to large sample size, multicollinearity was not detected.

We also controlled for race/ethnicity, insurance and income in the study. Race/ethnicity was categorized into non-Hispanic white (hereinafter White), non-Hispanic African American (hereinafter African American), Hispanic, and Asian/other. Insurance status categories included private insurance, Medicare, Medicaid, and uninsured. Annual individual income was categorized into three groups, less than $20,000, $20,000-$34999, and $35,000 or higher.

Further, based on established models and previous studies [4-6,34], we included other covariates representing demographic and socioeconomic characteristics - age, sex, education, marital status, employment status and region). Age was dichotomized into 18 to 64 and 65 or higher. Highest level of education was grouped into three categories-high school or less, high school diploma/general education development (GED), and bachelor and higher degree. Marital status was dichotomized into married and not married. Employment status was also dichotomized into employed and unemployed. Region includes four categories, northeast, midwest, south and west.

### Analyses

Descriptive analyses were first conducted to examine the unadjusted prevalence of each of the six indicators by health status and functional limitations, and among various racial/ethnic, insurance and income groups. Differences among groups were assessed using Chi-squared test. Logistic regression models were then built to examine the associations between access to care and the independent variables as well as the covariates.

## Results

### Descriptive and comparative statistics

Table 1 presents the unadjusted proportions of individuals who reported during the past 12 months having no usual place of care, inability to get medical care, having delayed medical care, being unable to get dental care, mental health care, or prescription drugs. Three most prevalent access issues were inability to get dental care, delayed medical care, and inability to get prescription drugs, with 15.34%, 14.78% and 12.03% of the sample reported having encountered each situation.

**Table 1:** Race/Ethnicity, insurance, income, and access to care: NHIS 2009

	**No usual place of care**		**Unable to get medical care**		**Delayed medical care**		**unable to get dental care**		**unable to get mental health care**		**unable to get prescription drug**	
	**%**	**n**	**%**	**n**	**%**	**n**	**%**	**n**	**%**	**n**	**%**	**n**
**Health status**	**	5463	***	5503	***	5502	***	5456	***	5456	***	5459
Total	5.14	270	9.98	626	14.78	850	15.35	891	4.05	216	12.03	693
Excellent/VG/Good	5.74	221	7.32	341	11.79	510	12.2	507	3.13	119	8.73	364
Fair/Poor	3.37	49	17.84	285	23.63	340	24.65	384	6.78	97	21.81	329
**Functional limitation**	***	5463	***	5503	***	5502	***	5456	***	5456	***	5459
Total	5.15	271	9.98	626	14.78	850	15.34	891	4.05	216	12.03	693
Yes	3.28	62	16.95	339	21.54	405	23.28	452	8.07	139	19.58	372
No	5.99	209	6.84	287	11.74	445	11.78	439	2.24	77	8.64	321
**Race/Ethnicity (%)**	**	5465	***	5505	***	5504	***	5458		5458	***	5461
Total	5.15	271	9.98	626	14.78	850	15.34	891	4.05	216	12.03	693
White	4.68	151	8.63	343	14.21	525	13.76	489	3.86	134	10.59	378
African American	5.30	44	16.32	154	18.49	163	21.19	208	5.73	41	18.14	169
Hispanic	8.88	62	14.91	111	17.69	135	22.19	159	4.22	32	18.19	122
Asian/Other	4.56	14	4.61	18	7.92	27	11.26	35	2.23	9	6.27	24
**Insurance (%)**	***	5241	***	5281	***	5280	***	5234	***	5234	***	5237
Total	5.16	260	9.84	592	14.66	808	14.85	824	4.05	205	11.84	658
Uninsured	27.84	115	38.87	193	50.87	227	48.18	191	16.87	60	40.23	169
Medicaid	4.37	18	14.23	75	17.88	89	26.96	135	7.42	36	19.28	101
Medicare	1.05	13	12.3	116	13.44	136	16.13	188	3.22	34	13.4	140
Private	3.56	114	5.57	208	10.30	356	9.17	310	2.33	75	7.26	248
**Income (%)**		2639	***	2651	***	2649	***	2638	**	2639	***	2639
Total	7.23	183	10.73	329	16.75	476	15.30	431	4.88	122	12.40	339
<$20,000	8.27	64	17.32	153	26.18	208	22.59	204	6.86	59	20.82	161
$20,000-$34,999	8.25	46	11.64	81	16.2	112	19.45	108	6.44	31	16.09	100
>=$35,000	6.21	73	6.53	95	11.5	156	9.38	119	3.09	32	6.02	78

Sample is limited to adult who visited a doctor or other health care professional during the past 2 weeks at the time of the survey.

*p<0.05, **p<0.01, ***p<0.001 based on χ^2^ test.

Respondents with fair or poor health status were less likely than those with good or better health to have no usual place of care (3.37% vs. 5.74%). However, significantly larger proportions of them reported being unable to get medical care, having delayed medical care, and being unable to get dental care, mental care, or prescription drugs. Similarly, respondents having functional limitations were less likely to have no usual place of care than those without functional limitations (3.28% vs. 5.99%), but more likely to have other access issues.

Significant differences were also observed by race/ethnicity, insurance status, and income level (Table 1). African Americans and Hispanics were significantly more likely than Whites and Asians to report having no usual place of care, being unable to get medical care, dental care or prescription drugs and having delayed medical care. Asians, however, more frequently reported having no usual place of care, but less frequently in other aspects.

In addition, those uninsured more frequently reported having no usual place of care (27.84%), delayed medical care (50.87%), being unable to get medical care (38.87%), dental care (48.18%), mental health care (16.87%), and prescription drugs (40.23%). Among the insured, Medicaid beneficiaries most frequently reported having difficulties obtaining care. Compared to Medicare beneficiaries, privately insured individuals reported having better access to medical care, dental care, mental health care, and prescription drugs, but a higher proportion of them reported having no usual place of care (3.56%).

Lastly, there is an inverse relationship between income level and difficulty obtaining care. Compared to individuals in higher income levels, those with annual income lower than $20,000 per year had the largest proportion of reporting inability to get medical care (17.32%), delayed medical care (26.18%), inability to get dental care (22.59%), mental health care (6.86%) or prescription drug (20.82%). The proportions were also significantly higher for individuals with annual income between $20,000 and $34,999 than those with annual income above $35,000.

### Logistic regressions

The influence of self-perceived health status and functional limitations on access was considerable after controlling for individual’s race/ethnicity, insurance status, income, and other covariates (Table 2). Those reporting fair or poor health status had more than two times higher odds of getting delayed medical care and being unable to get prescription drugs. Moreover, compared to those without functional limitations, those with limitations had two times higher odds of getting delayed medical care and being unable to get dental care, three times higher odds of being unable to get medical care and prescription drugs, and more than four times higher odds of being unable to get mental health care.

**Table 2:** Logistic regressions of predictors associated with access to care: NHIS 2009

	**Odds ratio (95% confidence interval)**					
	**No usual place of care**	**Unable to get medical care**	**Delayed medical care**	**Unable to get dental care**	**Unable to get mental health care**	**Unable to get prescription drug**
**Self-perceived health status**						
Excellent/VG/Good (reference)	1	1	1	1	1	1
Fair/Poor	0.831 (0.433 1.595)	1.43 (0.913 2.24)	2.221*** (1.436 3.435)	1.532 (0.949 2.473)	0.728 (0.333 1.59)	2.038** (1.284 3.235)
**Functional limitation**						
Yes	0.537 (0.264 1.094)	3.043*** (1.917 4.831)	2.049*** (1.365 3.075)	2.226** (1.366 3.625)	4.371*** (2.395 7.977)	3.08*** (1.933 4.91)
No (reference)	1	1	1	1	1	1
**Race/Ethnicity**						
White (reference)	1	1	1	1		1
African American	0.709 (0.304 1.652)	1.764* (1.027 3.03)	0.822 (0.474 1.427)	0.921 (0.515 1.647)	0.897 (0.355 2.267)	1.161 (0.659 2.048)
Hispanic	1.226 (0.666 2.254)	1.308 (0.722 2.37)	0.612 (0.351 1.067)	1.26 (0.769 2.067)	0.581 (0.302 1.119)	0.917 (0.477 1.761)
Asian/Other	1.176 (0.462 2.99)	1.334 (0.68 2.618)	0.636 (0.331 1.224)	1.145 (0.553 2.372)	1.221 (0.422 3.532)	0.676 (0.362 1.262)
**Insurance**						
Uninsured	14.151*** (7.161 27.963)	7.934*** (4.796 13.125)	8.319*** (4.843 14.29)	5.996*** (3.415 10.526)	5.35** (1.744 16.412)	3.291*** (1.747 6.198)
Medicaid	3.411* (1.161 10.022)	1.804 (0.811 4.012)	1.402 (0.606 3.243)	2.222* (1.007 4.906)	2.708 (0.693 10.583)	1.978 (0.875 4.473)
Medicare	1.058 (0.804 2.682)	4.437*** (1.989 9.899)	2.754* (1.185 6.399)	2.058 (0.904 4.682)	2.157 (0.702 6.632)	2.002 (0.839 4.78)
Private (reference)	1	1	1	1	1	1
**Income**						
<$20,000	0.537* (0.296 0.973)	0.851 (0.541 1.34)	1.103 (0.705 1.725)	0.956 (0.572 1.597)	1.113 (0.427 2.903)	1.043 (0.605 1.799)
$20,000-$34,999 (reference)	1	1	1	1	1	1
>=$35,000	0.916 (0.473 1.775)	0.849 (0.485 1.488)	0.987 (0.609 1.601)	0.805 (0.498 1.299)	1.474 (0.559 3.888)	0.608 (0.341 1.083)
**Age**						
18-64	6.966* (1.189 40.797)	1.576 (0.798 3.11)	3.327** (1.617 6.845)	1.521 (0.775 2.986)	2.395 (0.962 5.964)	2.502* (1.224 5.117)
65+ (reference)	1	1	1	1	1	1
**Sex**						
Female	0.637 (0.375 1.081)	1.235 (0.822 1.854)	1.13 (0.794 1.608)	1.076 (0.747 1.549)	0.8 (0.45 1.424)	1.137 (0.752 1.718)
Male (reference)	1	1	1	1	1	1
**Education**						
High School or less	1.137 (0.554 2.334)	0.812 (0.462 1.425)	0.853 (0.52 1.401)	1.243 (0.753 2.053)	1.037 (0.481 2.232)	1.059 (0.61 1.838)
High School Diploma/ GED (reference)	1	1	1	1	1	1
Bachelor and Higher Degree	1.419 (0.775 2.596)	0.771 (0.465 1.279)	0.825 (0.554 1.228)	0.978 (0.639 1.498)	0.734 (0.327 1.647)	0.561* (0.346 0.911)
**Marital status**						
Married (reference)	1	1	1	1	1	1
Not Married	1.774* (1.038 3.032)	2.031*** (1.362 3.028)	2.067*** (1.438 2.972)	1.822** (1.249 2.659)	2.443** (1.292 4.616)	1.553* (1.028 2.346)
**Employment status**						
Employed (reference)	1	1	1	1	1	1
Not Employed	1.977* (1.087 3.596)	0.69 (0.417 1.142)	1.33 (0.815 2.171)	0.694 (0.423 1.139)	1.017 (0.513 2.016)	0.946 (0.606 1.478)
**Region**						
Northeast	0.724 (0.35 1.5)	0.787 (0.462 1.343)	0.841 (0.521 1.358)	0.556* (0.318 0.972)	0.476 (0.207 1.095)	0.603 (0.336 1.08)
Midwest	0.848 (0.396 1.816)	1.003 (0.57 1.763)	1.03 (0.651 1.63)	0.873 (0.552 1.382)	0.589 (0.275 1.262)	0.543* (0.308 0.956)
South (reference)	1	1	1	1	1	1
West	1.141 (0.548 2.376)	1.466 (0.839 2.561)	2.101** (1.341 3.292)	1.173 (0.718 1.917)	0.499 (0.201 1.24)	0.736 (0.45 1.206)

Sample is limited to adults who visited a doctor or other health care professional during the past 2 weeks at the time of the survey.

*p<0.05, **p<0.01, ***p<0.001.

Furthermore, after controlling for health status and socioeconomic characteristics, disparities in access to care due to race/ethnicity and income level became less significant. Differences between Whites and Hispanics were insignificant in all six attributes. African Americans have similar patterns as Whites except for being unable to get medical care for which African Americans have almost 77% higher odds. The odds of delayed medical care was nearly 65% lower for Asians than for Whites. In terms of income, compared to individuals with annual income between $20,000 and $34,999, individuals with lower income level had 47% reduced odds of having no usual place of care.

On the other hand, insurance status became the single factor that was associated with all six measures of access to care after adjusting for other factors. Disparities between uninsured and privately insured were the most significant. Uninsured had more than fourteen times higher odds of having no usual place of care, around eight times higher odds of being unable to get medical care or delayed medical care, almost six times higher odds of being unable to get dental care, more than five times higher odds of being unable to get mental health care, and more than three times higher odds of being unable to get prescription drugs. In addition, compared to private insurance holders, Medicaid beneficiaries had about 3.4 times greater odds of reporting no usual place of care and 2.2 times greater odds of reporting inability to get dental care. Furthermore, Medicare recipients had around 4.5 times higher odds of reporting being unable to get medical care and 2.8 times higher odds of delayed medical care than adults covered by private insurance. However, the odds of having no usual place of care for Medicare beneficiaries was much lower (<0.001) than for adults covered by private insurance.

## Discussion

Health status and functional limitations are closely associated with access to care. The presence of functional limitations, a common measure of disability and multimorbidity, is associated with five of the six measures of access. People with poorer health experience more access barriers even after controlling for insurance and other measures. This finding indicates that interventions to enhance access should target those with greatest need, i.e., people with poorer health status.

Furthermore, although a large number of studies have established that racial/ethnic disparities persist in the United States, our study found that after controlling for insurance and health status, as well as other socioeconomic factors, differences between Whites and racial/ethnic groups in most measures of access were not significant. In fact, previous research has observed that disparities by race/ethnicity were more “nuanced” than was typically described in the literature [17]. Income was also found to be not significantly related to inadequate care. Alternatively, insurance was found to be an important factor that was associated with all six measures of access to care. Uninsured populations were significantly more likely to have no usual place of care and difficulties getting care. One of the main goals of the Affordable Care Act is to promote universal coverage by provisions such as expanding Medicaid and establishing State Health Insurance Exchanges. Based on our study, these measures to expand insurance coverage are expected to further improve access to care and reduce racial/ethnic disparities. However, among those insured, Medicaid beneficiaries were more likely to report having no usual place of care and inability to get dental care, while Medicare recipients more frequently reported inability to get medical care and delayed medical care, indicating that there are still gaps in benefits between public and private insurance. Further, the current health care system may not be able to quickly adapt to meet the increasing demands from the newly covered. Therefore, expansion in insurance should be accompanied with improvements in public insurance system and enhancement of the health care system [18,23,39].

Nevertheless, there are limitations in our study. First, due to the cross-sectional nature of the survey, we could not examine the causal relationships between access to care and potential predictors. Secondly, since data on income, health status, functional limitations, etc. were self-reported, bias may exist. Thirdly, because of data availability, other important factors such as health care system characteristics, external environment and language barriers were not taken into consideration. Lastly, by only including respondents who visited a health provider in the previous two weeks, the generalizability of the findings may be compromised. Future studies should also focus on quality of care received by different racial/ethnic and insurance groups and its ultimate effects on health outcomes.

In conclusion, using the latest data, this study found that disparities in access to care among racial/ethnic groups as well as income levels have been reduced significantly. Insurance and health status (in particular functional limitations) were found to be the most important factors that were associated with access to care. Therefore, interventions to expand insurance coverage and the Affordable Care Act are expected to contribute to reducing disparities in access to care. However, to further improve access to care, targeted interventions and assistance must be given to those who are of poorer health status.

## Competing interests

The authors declare that they have no competing interests.

## Authors’ contributions

LS conceptualized the study, TW and JZ carried out the analyses, XN drafted the manuscript. All authors read and approved the final manuscript.
